# Toll‐like receptor 9 signaling in chronic lymphocytic leukemia cell lines

**DOI:** 10.1002/2211-5463.13726

**Published:** 2023-11-08

**Authors:** Miriam Meloni, Ilenia Sana, Maria Elena Mantione, Michela Riba, Marta Muzio

**Affiliations:** ^1^ Cell Signaling Unit, Division of Experimental Oncology San Raffaele Hospital IRCCS Milan Italy; ^2^ Center for Omics Sciences San Raffaele Hospital IRCCS Milan Italy

**Keywords:** cell line, chronic lymphocytic leukemia, CpG, NFKBIZ, Toll‐like receptor 9

## Abstract

Chronic lymphocytic leukemia (CLL) is a prototypic neoplasia in which malignant cells strongly depend on microenvironmental stimulations in the lymphoid tissues where they accumulate; leukemic cells are exposed to interaction with bystander and accessory cells, as well as inflammatory soluble mediators. Cell lines are frequently used to model the pathobiology of this disease; however, they do not always recapitulate leukemic cell growth and response to stimulation, and no data are available on Toll‐like receptors (TLR) signaling in CLL cell lines. To address this gap, we analyzed HG3, MEC2, and PCL12 cell lines, before and after CpG stimulation, by RNA‐sequencing followed by bioinformatic analyses and validation experiments. We identified NFKBIZ mRNA and the corresponding IkBz protein as robust markers of TLR9 activation in both MEC2 and PCL12, but not in HG3 cells. Next, we compared our current results with previous results obtained with primary CLL patient samples and were able to conclude that MEC2 is most similar to the patients' cells in terms of global responsiveness to TLR stimulation; in particular, MEC2 better resembles the samples of patients, as it is characterized by high expression levels of IkBz, but with a lower number of genes regulated.

## Graphical abstract

To model the pathobiology of TLR9 response in CLL cell lines, we analyzed the transcriptome of MEC2, PCL12, and HG3 after CpG stimulation. MEC2 best recapitulates the characteristics of primary CLL cells in terms of TLR9 signaling, IkBz expression, and global molecular fingerprint, pointing to this cell line as the most appropriate preclinical model to study TLR stimulation in CLL.

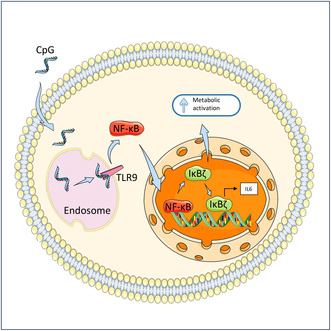

**Abbreviations**
BcR: B‐cell receptor
CLL: chronic lymphocytic leukemia
DEGs: differentially expressed genes
FC: fold change
FDR: false discovery rate
GSEA: gene set enrichment analysis
IGHV: immunoglobulin heavy variable
NES: Normalized Enrichment Score
TLR: Toll‐Like receptor

Monoclonal malignant B‐lymphocytes accumulate in the peripheral blood, bone marrow, and secondary lymphoid organs of patients with chronic lymphocytic leukemia (CLL). These cells maintain several features of normal B‐lymphocytes, including the expression of a clonotypic B‐cell Receptor (BcR) and of distinct innate immune Toll‐like receptors (TLR) [1]. On the contrary, CLL cells invariably express on their surface CD5, a prototypic T‐cell specific protein ‘aberrantly’ expressed by leukemic cells and representing a diagnostic marker together with CD19, CD20, and CD23 [2]. CLL accounts for 1% of all new cancer cases, and it is one of the most common adult leukemia affecting mainly the elderly with an average age at first diagnosis of 70 years (SEER Cancer stat facts; https://seer.cancer.gov/statfacts/html/clyl.html). Despite significant progress in the identification and optimization of novel treatment strategies, profound responses can be obtained in a minority of patients, and novel approaches as well as model systems are urgently needed.

Chronic lymphocytic leukemia is a very heterogeneous disease where distinct prognostic factors have been identified, including specific genetic abnormalities [1]; moreover, the somatic hypermutation status of the immunoglobulin heavy variable (IGHV) genes within the leukemic cells helps to refine risk stratification of the patients [3, 4]. Notably, CLL is a prototypic microenvironment‐dependent tumor where leukemic cells co‐evolve with accessory cells into an inflammatory milieu [5]; among several cytokine receptors, TLRs have been implicated in cell proliferation and survival in human primary cells and samples (6, 7, 8, 9, 10, 11) but not in a transgenic mouse model of leukemia [12]. Previous studies demonstrated the correlation between the induction of the atypical transcription factor IkBz, mediated by TLR9 stimulation, and a more active cellular response; accordingly, patients' samples were classified as IkBz‐low or IkBz‐high that show increased metabolic activity and resistance to chemotherapy [13]. This highlights the need for careful characterization of this signaling framework in primary leukemic cells as well as in human cell lines.

Cell lines are frequently used to model the pathobiology of tumors, including leukemia and lymphoma. However, CLL is somewhat an exception as few cell lines have been stabilized and characterized likely due to the intrinsic difficulty to immortalize circulating malignant cells. Among them, MEC1/MEC2, PCL12, and HG3 are the most frequently used; they express a clonotypic BcR bearing either mutated (MEC1 and MEC2) or unmutated (PCL12 and HG3) IGHV genes, thus representing the two different subgroups of CLL cases. CD5 can be expressed by HG3 and PCL12, but it decreases over prolonged *in vitro* culture; CD19 and CD20 are expressed in all the three cell lines, while CD23 is expressed at lower levels in MEC2 as compared to HG3 and PCL12 [14, 15, 16, 17]. Overall, these data suggest that CLL cell lines are useful preclinical models even if they are not identical to their primary counterpart. Accordingly, they have been widely used for drug response as well as for BcR signaling studies [16, 17, 18]. However, no data are available on their TLR‐mediated signaling capabilities as compared to primary leukemic cells. To address this issue, we analyzed the transcriptomes of these three different cell lines treated *in vitro* with CpG, and we compared their gene expression profiles with that of primary CLL cells previously analyzed [19].

## Methods

### Cell culture and treatment

The EBV‐positive MEC1, MEC2, PCL12, and HG3 cell lines were purchased from DMSZ and cultured with RPMI medium (Gibco, Waltham, MA, USA) supplemented with 10% heat‐inactivated FBS (Gibco) and Penicillin/Streptomycin (100 U·mL^−1^; Invitrogen, Waltham, MA, USA). ODN‐2006 CpG‐class B oligonucleotide specific for human TLR9 (InvivoGen, San Diego, CA, USA) was added to the cells at a final concentration of 2.5 μg·mL^−1^ as previously described for primary CLL cells [13].

### RNA‐sequencing and analysis

Total RNA was extracted from unstimulated or stimulated MEC2, PCL12, and HG3 cells (4 h with CpG) using the Qiagen RNA extraction kit (Hilden, Germany). The library was prepared using a stranded reverse protocol. Libraries have been checked using Qubit (fluorimeter) and Bioanalyzer (capillary electrophoresis). Sequencing was performed using Illumina Novaseq S2 XP, 1 × 100 nt, single read (Illumina, San Diego, CA, USA). Both library preparation and RNA‐sequencing were performed in the Genomics Laboratory of the Center for Omics Science (COSR), San Raffaele Hospital, Milano, Italy. The trimmed sequences obtained were aligned to the human ‘Hg38’ genome. RNA‐seq raw data have been deposited in NCBI's Gene Expression Omnibus [20] and are accessible through GSE233595.

Putative differentially expressed genes (DEGs) between CpG‐stimulated and unstimulated samples were selected using limma [21]. Genes are indicated as ‘Differentially Expressed Genes’ (DEGs) if they satisfy both the following conditions: nominal *P* value < 0.01 and |Log_2_FoldChange| ≥ 1 (|Log_2_FC|).

Volcano plots were prepared using the ggplot2 package r (https://CRAN.R‐project.org/package=ggplot2).

### Real‐time PCR

Total RNA was extracted from unstimulated or stimulated cells (4 and 24 h with CpG) using the RNeasy Plus Mini kit (Qiagen) and was quantified with nanodrop, and 1 μg was retro‐transcribed with iScript Advanced cDNA Synthesis kit (Bio‐Rad, Segrate, Italy).

cDNA was used as template for real‐time PCR using CFX Connect Real‐Time PCR Detection System (Bio‐Rad) with the Probe protocol and the following probes:

Probe NFKBIZ (qHsaCEP0052487; Bio‐Rad).
Probe IL6 (Hs00985639m1; Thermo Fisher Scientific, Waltham, MA, USA).
Probe β‐Actin (qHsaCEP0036280; Bio‐Rad).
Probe TBP (qHsaCIP0036255; Bio‐Rad).

Relative mRNA expression levels of NFKBIZ and IL6 were calculated using the $2^{-\Delta \Delta C_{t}}$ and $2^{-\Delta C_{t}}$ methods, respectively, with β‐Actin and TBP as endogenous reference genes.

Statistical analysis was performed with prism Software (graphpad prism 10 Software, Boston, MA, USA) as indicated in each figure legend.

### Western blot

One million cells were lysed in 60 μL of RIPA buffer and centrifuged, and the supernatant was added to reducing agent and loading buffer; after boiling, the samples were loaded onto an SDS/PAGE gel (precast gels 4–12% acrylamide; Invitrogen), run, and immobilized onto a nitrocellulose membrane (The Trans‐Blot Turbo Transfer System). Anti‐IkBz antibody #9244 (Cell Signaling Technology), anti‐phospho‐ERK1/2, p44/42 MAPK #9104 (Cell Signaling Technology, Danvers, MA, USA), and anti‐ERK1/2, p44/42 MAPK SC‐514302 (Santa Cruz Biotechnology, Dallas, TX, USA) were added to the membranes; after addition of the secondary HRP‐conjugated antibody A0545 (Sigma‐Aldrich, Schnellendorf, Germany) or and anti β‐Actin antibody HRP‐conjugated, 8H10D10 (Cell Signaling Technology), and the ECL detection reagent, the images were acquired with the ChemiDoc MP Imaging System (Bio‐Rad).

Densitometric analysis was performed utilizing the image lab software (Bio‐Rad). Statistical analysis was performed with prism Software (graphpad prism 10 Software) as indicated in each figure legend.

## Results and Discussion

### Transcriptome analysis of chronic lymphocytic leukemia cell lines

To explore TLR9‐mediated signaling pathways in CLL cell lines, we performed a transcriptome analysis, including coding and noncoding RNAs in HG3, MEC2, and PCL12 cells treated *in vitro* with the immunostimulatory TLR9 agonist ODN‐2006, a class B unmethylated CpG oligonucleotide specific for human cells. Four hours after incubation, the cells were washed and pelleted and the RNA was purified for sequencing.

**Fig. 1:**
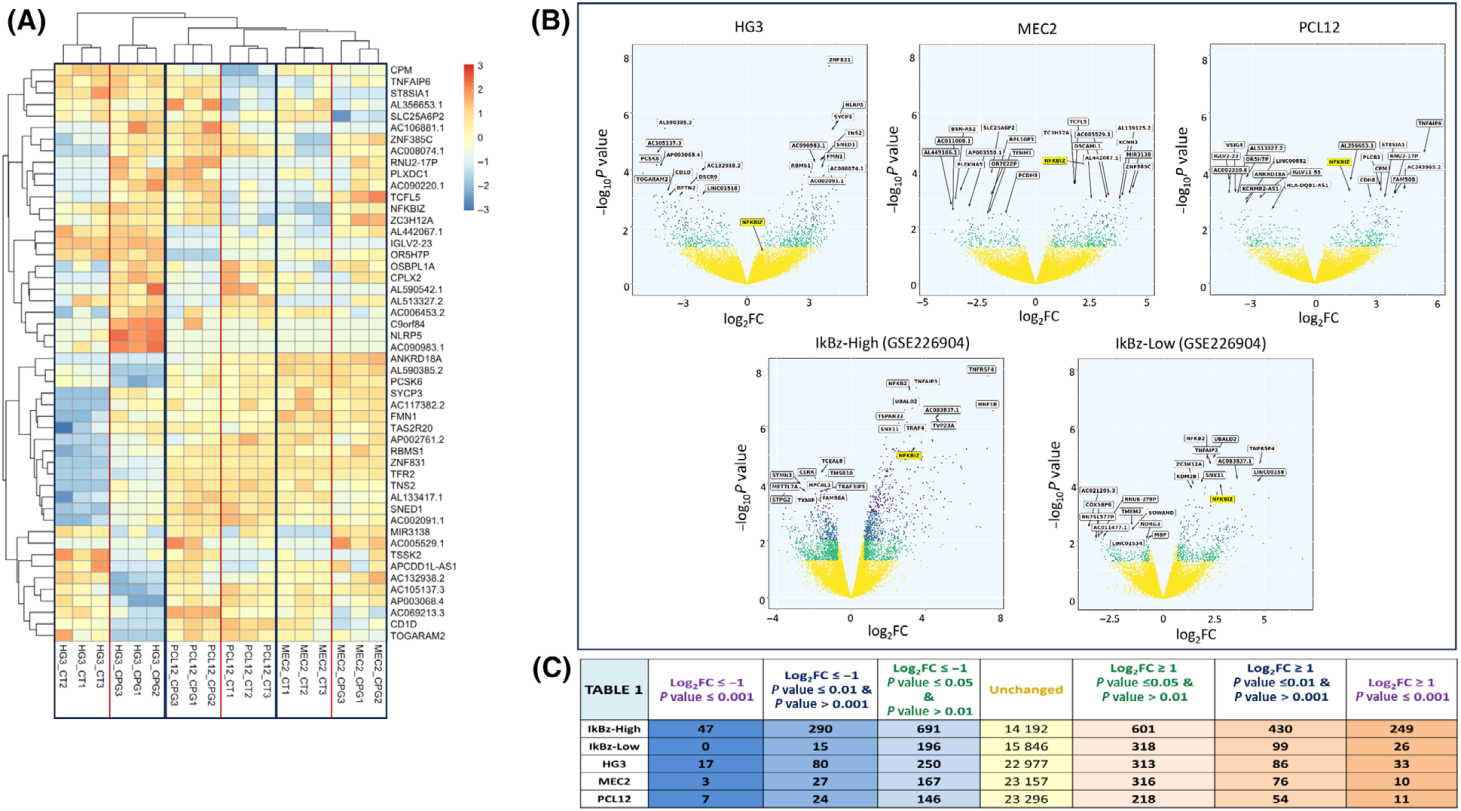
Heat map and Volcano plot of transcriptome analysis of the cell lines. (A) Heat map showing the clustered expression values of 50 DEGs between CpG‐treated and untreated cell lines. (B) Volcano plots of HG3, MEC2, and PCL12 as compared to GSE226904; the *X*‐axis denotes the values of Log_2_FC and the *Y*‐axis the values of Log_10_ *P* value. Significant genes are defined by |Log_2_FC| ≥ 1 and different *P* values: *P* value between ≤ 0.05 and > 0.01 (green); *P* value between ≤ 0.01 and > 0.001; (blue); *P* value ≤ 0.001 (violet). Unchanged genes are represented in yellow. (A) Table reporting the number of genes analyzed and differentially expressed is reported for each cell line as well as patient's samples as a comparison (Panel C).

Heat map shows the clustered expression values of 50 DEGs between CpG‐treated and untreated cell lines (Fig. 1A); this clustering suggests that HG3 cell line is quite distant from the other two and that a limited number of genes are differentially expressed in stimulated vs unstimulated samples in all the three cell lines.

All the expressed genes were graphically represented with a Volcano plot that shows global perturbation of the transcriptomes, including up‐ and downregulated genes (Fig. 1B); as a comparison, we reported the same graph with previously analyzed data of primary CLL patients' samples, including IkBz‐high and IkBz‐low cases (GSE226904) [19]. Volcano plots show an overall increased number of DEGs, as genes with nominal *P* value < 0.01 and |Log_2_FC| ≥ 1, in IkBz‐high CpG samples as compared to both IkBz‐low and CLL cell lines. Specifically, as reported in Fig. 1C, HG3 had a total of 432 genes upregulated and 347 genes downregulated; MEC2 and PCL12 had a less reactive profile with 402 and 283 genes upregulated and 197 and 177 genes downregulated, respectively.

Top 10 DEGs both upregulated and downregulated in each cell line are indicated with their official gene symbol in each plot above the yellow area; among them, the NFKBIZ gene encoding for IkBz protein emerged in two out of the three cell lines analyzed (MEC2 and PCL12) with a stringent *P* value ≤ 0.001 and |Log_2_FC| ≥ 1. In contrast, NFKBIZ was not significantly upregulated in HG3 (highlighted below the yellow area in the relative plot; Fig. 1B).

Intersection of the list of DEGs showed no common genes shared by all the 3 cell lines; however, a limited set of common genes was observed in selected pairs. In detail, both HG3 and MEC2 upregulate ZNF385C and OSBPL1A and downregulate CD1D. The HG3 and PCL12 cell lines co‐upregulate AC106881.1, PLXDC1, and AC090220.1. MEC2 and PCL12 share the upregulation of ZC3H12A, AL139011.1, and NFKBIZ. Among all the upregulated noncoding and protein‐coding genes, NFKBIZ was the only one to be previously described as induced by different TLRs, including TLR9 [13, 22]. To note, ZC3H12A (MCPIP1) is an endoribonuclease that acts as feedback inhibitor of IL17 signaling by favoring the degradation of different inflammatory mRNAs, including NFKBIZ in T‐cells [23]. These data suggest that ZC3H12A may be involved in a feedback loop of TLR9 signaling; however, in our cellular models, we could detect the transcripts of both NFKBIZ and its ‘negative regulator’ ZC3H12A at the same time.

Overall, from this gene expression analysis it is difficult to identify the cell line most similar to the patients' samples in terms of TLR9 stimulation as quite few genes are shared. Even if the HG3 cell line showed a higher number of DEG, it failed to upregulate NFKBIZ that is the only bona fide TLR‐response gene of the list [22].

### CpG‐induced IkBz expression in the cell lines

**Fig. 2:**
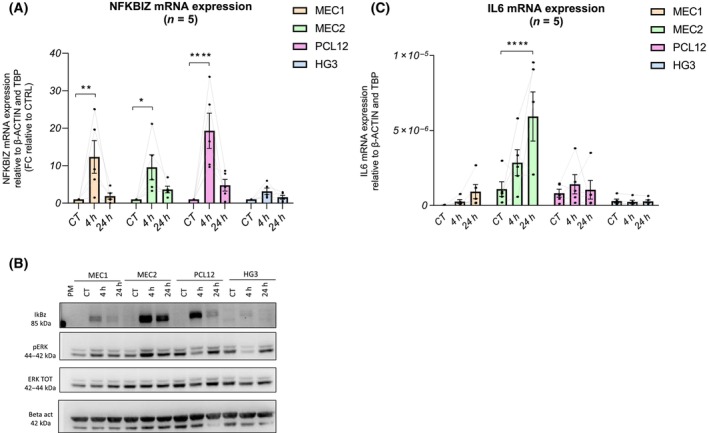
NFKBIZ mRNA and IkBz protein expression in cell lines. MEC1, MEC2, PCL12, and HG3 cell lines were treated with CpG for 4 and 24 h and a real‐time PCR analysis was performed for NFKBIZ (A) and IL6 (C) expression. Data are reported as relative mRNA expression calculated with $2^{-\Delta \Delta C_{t}}$ and $2^{-\Delta C_{t}}$ methods, respectively, with β‐Actin and TBP as endogenous reference genes. All the data in Panels A and C are reported as relative expression levels (mean ± SD). Two‐way ANOVA was performed followed by Dunnett's multiple comparisons test for each cell line compared with their respective controls; *P value < 0.05; **P value < 0.01; ****P value < 0.0001. (B) One representative experiment out of four is reported where the cell lines were stimulated with CpG at different time points (4 and 24 h) and IkBz protein expression was assessed by western blot analysis. β‐actin was used as loading control. Phospho‐ERK (pERK) and total ERK were analyzed in the same samples as indicated.

In order to validate NFKBIZ expression and regulation in the CLL cell lines, we performed real‐time PCR analysis at different time points after CpG stimulation (4 and 24 h). First, we confirmed that low/no induction of NFKBIZ was detected in HG3 cells. A statistically significant induction was observed in MEC2 and PCL12 cells treated with CpG for 4 h (Fig. 2A). We added to this analysis also MEC1 cell line that was isolated from the same patient of the MEC2 and shares several similar characteristics [17]. MEC1 cells were also responsive in terms of NFKBIZ mRNA induction similar to the sister MEC2.

Previous data of primary CLL patients' samples (both IkBz‐high and IkBz‐low) reported a similar pattern of NFKBIZ mRNA induction at early time points that decreased after 24 h [13]. However, at protein levels, only the IkBz‐high samples maintained IkBz induction after 24 h of TLR9 stimulation. Therefore, we performed western blot analysis to investigate whether and which cells maintained the capacity to induce stable IkBz protein. As shown in Fig. 2B, IkBz protein is not detectable in unstimulated cells while it is induced after CpG addition to the cell culture. In detail, MEC2, and to a lesser extent PCL12, expressed IkBz already at 4 h, and it was stable up to 24 h (Fig. 2B shows one representative experiment out of four). MEC1 cells produced low levels of IkBz at 4 h, but the protein was almost undetectable at 24 h. In contrast, HG3 cells did not show any IkBz protein induction (Fig. 2B).

Next, we analyzed ERK activation as it was previously shown to be constitutively activated in MEC1 and PCL12 cells as well as in a group of CLL patients' cells rendering these cells anergic toward BcR stimulation [24]. ERK was constitutively phosphorylated in all the cell lines analyzed. When MEC2 cells were stimulated with CpG, induction of phospho‐ERK was detected at early time points in three out of four independent experiments (Fig. 2B and data not shown). MEC1 cells were only partially responsive in terms of ERK phosphorylation. On the contrary, ERK phosphorylation was reduced in PCL12 and HG3 cells after 4 h of TLR9 stimulation in all the four replicates returning to basal levels after 24 h (Fig. 2B and data not shown).

Overall, these data suggest that basal levels of ERK phosphorylation do not impede TLR9 signaling in MEC2 and to a lesser extent MEC1; surprisingly, in HG3 and PCL12 TLR9 stimulation was effective but opposite and resulted in decreased levels of ERK phosphorylation.

IkBz is an atypical co‐transcription factor that controls inflammatory genes that are induced during the so‐called secondary inflammatory response, including the pro‐inflammatory cytokine interleukin‐6 (IL6). Indeed, in CLL cells it can be induced by CpG after IkBz upregulation [25].

Transcriptomic analysis confirmed the significant induction of IL6 mRNA in MEC2 and PCL12 but not in HG3 cells (Log_2_FC = 2.31 with *P* value = 0.02 for MEC2; Log_2_FC = 3.05 with *P* value = 0.004 for PCL12).

To monitor IL6 mRNA levels over time, we performed real‐time PCR analysis in MEC1, MEC2, PCL12, and HG3 cells before and after CpG stimulation. To note, PCL12 and MEC2 expressed higher basal levels of IL6 mRNA as compared to the others; however, MEC2 only responded to CpG with highly variable levels of IL6 mRNA induction at 24 h (Fig. 2C).

This biochemical analysis suggested that MEC2 and PCL12 but not HG3 may resemble primary CLL samples, in particular the IkBz‐high subgroup.

### MEC2 cells resemble primary IkBz‐high CLL samples

**Fig. 3:**
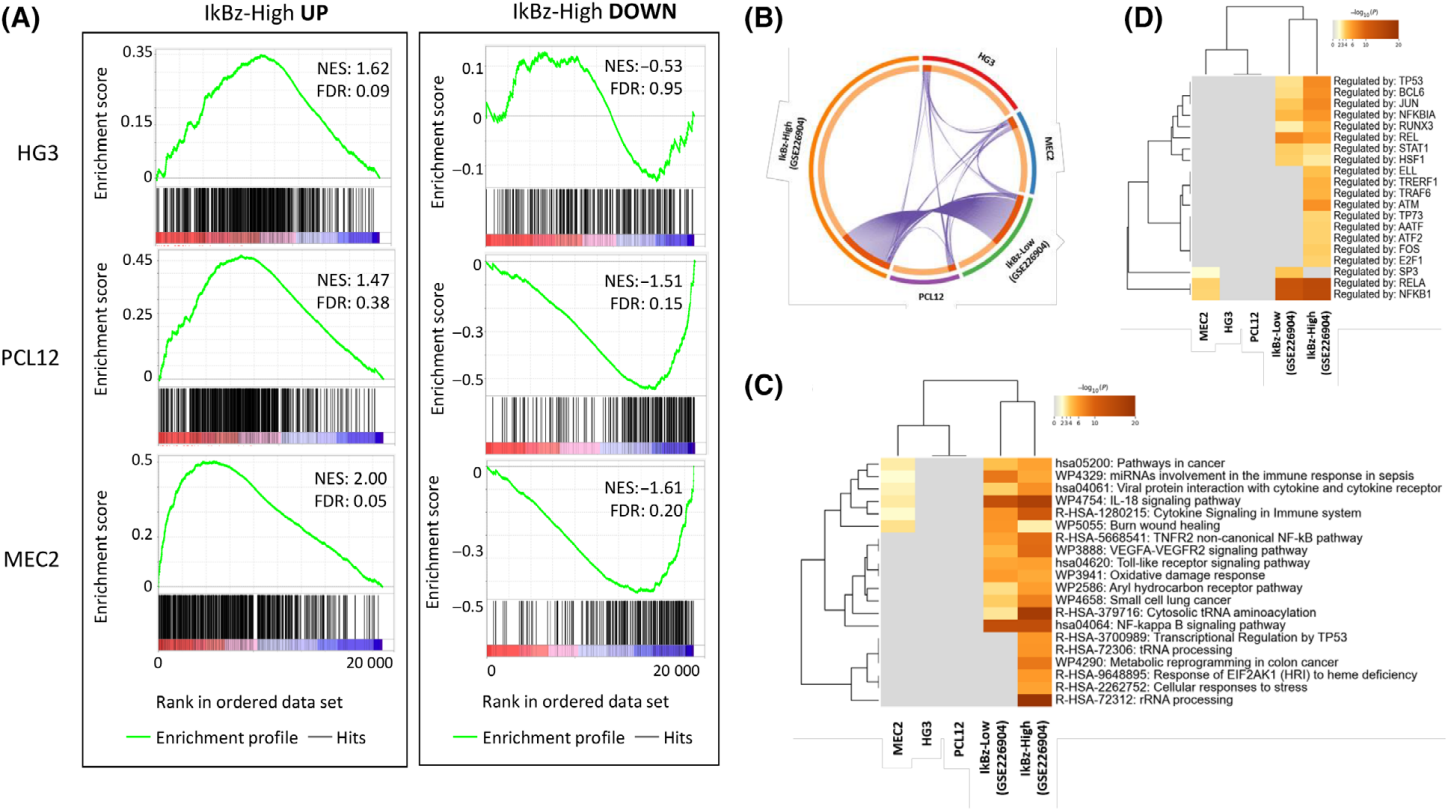
Gene Set Enrichment Analysis (GSEA) and Metascape analysis of DEGs. (A) We created specific gene sets from lists of DEGs in IkBz‐high samples (GSE226904), and we used them to perform enrichment analysis of the cell line transcriptomes. Enrichment plots for each cell line against upregulated (UP) or downregulated (DOWN) gene sets are reported as indicated. In each panel, the green curve represents the running enrichment score and the black bars indicate the positions of the gene set hits on the rank‐ordered list in GSEA. The highest signal‐to‐noise values are indicated with red color, while blue indicates the lowest ones. Significant gene sets are defined by |NES| ≥ 1.5 and FDR ≤ 0.25 shown inside the plot. For paired contrast ‘CpG‐stimulated vs ctrl’, we calculated the DEGs as genes with |Log_2_FC| ≥ 1 and nominal *P* value ≤ 0.05, and with those lists, we performed enrichment analysis with Metascape. (B) Circos plot showing the DEGs overlapping among the lists of upregulated genes. (C) Heat map showing the top 20 enriched pathways among the lists of DEGs identified in the meta‐analysis. The heat map box is colored by their *P* values, and gray box indicates the lack of enrichment for that term in the corresponding gene list against KEGG, REACTOME, and canonical pathways. (D) Enrichment analysis against transcriptional regulatory network collection of gene set TRRUST was performed, and the heat map shows the enriched terms among the lists of DEGs identified in the meta‐analysis for each cell line and the GSE226904 patients' samples as indicated.

To understand whether and which cell lines better represent primary patients' samples at global level, in terms of TLR9 signaling, we compared the transcriptome profiles of HG3, MEC2, and PCL12 with the ones of patients' leukemic cells (GSE226904). To overcome the limitation of the small size of the lists of DEGs to compare, we exploited GSEA (Gene Set Enrichment Analysis) [26, 27] to extrapolate distinct gene sets from our patients' data and perform a comparative analysis that could embrace overall concordance of response to TLR9 stimulation beside the amplitude of each single FC. We extracted from our patients' gene expression profiles (GSE226904) four different gene sets listing the upregulated and downregulated genes in both IkBz‐high and IkBz‐low samples. Next, we compared the cell line profiles of control and stimulated samples. This examination resulted in more significant values of similarity for MEC2 as assessed by high Normalized Enrichment Score (NES) and low false discovery rate (FDR); when compared to IkBz‐high upregulated genes, MEC2 showed a NES = 2 and FDR = 0.05 while HG3 was the less represented (Fig. 3A).

To further enlarge the spectrum of analysis and to capture subtle similarities, we selected genes with a |Log_2_FC| ≥ 1 and a *P* value < 0.05 and we performed a comparative analysis of both cell lines and patients' samples. The three cell lines shared only a limited number of genes represented by the purple lines in the Circos plot (Fig. 3B). Next, to explore the global perturbations in terms of TLR9‐mediated signaling activation, we performed pathway enrichment analysis with Metascape [28]. Metascape allows to perform enrichment analysis of multiple gene lists at the same time with the advantage of performing a concomitant comparative analysis of all the results. In particular, the investigation of the pathways involved showed that MEC2 cells are the only ones sharing some similarity with both categories of patients' samples, including different inflammatory/cytokine pathways (Fig. 3C). As expected, the two groups of primary leukemic cells share an inflammatory response, but IkBz‐positive samples only show stress‐related and RNA metabolism signatures [19].

In parallel, the enrichment analysis was performed against TRRUST (Transcriptional Regulatory Relationships Unraveled by Sentence‐based Text mining) curated gene sets, including gene lists regulated by specific transcription factors; this examination resulted in MEC2 as the only profile shared with CLL primary samples. Genes regulated by NFKB1 and RELA prototypic inflammatory factors are enriched in MEC2 and CLL (both subgroups) while SP3 emerged in MEC2 and IkBz‐low samples (Fig. 3D).

In conclusion, MEC2 and PCL12 cell lines showed a dampened response to TLR9 stimulation as compared to primary patients' samples. On the contrary, HG3 showed a higher number of DEGs; however, from pathway analysis they appeared very distant from primary leukemic cells. Overall, among the cell lines analyzed, MEC2 cell line best recapitulates the characteristics of primary CLL patient cells in terms of TLR9 signaling and IkBz expression; however, we cannot propose them as a ‘representative cell line’ for all the patient cells. Notwithstanding the lower response of MEC2 in terms of DEGs, advanced enrichment analysis called attention to a similar molecular fingerprint that pointed to this cell line as the most appropriate preclinical model to study TLR stimulation in CLL.

## Conflict of interest

The authors declare no conflict of interest.

### Peer review

The peer review history for this article is available at https://www.webofscience.com/api/gateway/wos/peer‐review/10.1002/2211‐5463.13726.

## Author contributions

MiM and IS designed the research, performed the experiments and bioinformatic and statistical analysis, and wrote the manuscript; MEM participated in experimental planning and performed experiments and data analysis; MR analyzed the RNA‐sequencing data and supervised the bioinformatic analyses; MaM designed and supervised the research and wrote the manuscript.

## Data Availability

The RNA‐seq data that support the findings of this study are openly available in Gene Expression Omnibus at (https://www.ncbi.nlm.nih.gov/geo/), reference number GSE226904 and GSE233595. The other data that support the findings of this study are available from the corresponding author (muzio.marta@hsr.it) upon reasonable request.
